# Behavioral effects of triazolam and pregnanolone combinations: reinforcing and sedative-motor effects in female rhesus monkeys

**DOI:** 10.3389/fpsyt.2023.1142531

**Published:** 2023-05-12

**Authors:** Jemma E. Cook, Donna M. Platt, Daniela Rüedi-Bettschen, James K. Rowlett

**Affiliations:** Department of Psychiatry and Human Behavior, Center for Innovation and Discovery in Addictions, University of Mississippi Medical Center, Jackson, MS, United States

**Keywords:** benzodiazepine, neuroactive steroid, self-administration, sedation, rhesus monkey (*Macaca mulatta*)

## Abstract

**Introduction:**

Benzodiazepines (BZs) are prescribed as anxiolytics, but their use is limited by side effects including abuse liability and daytime drowsiness. Neuroactive steroids are compounds that, like BZs, modulate the effects of GABA at the GABA_A_ receptor. In a previous study, combinations of the BZ triazolam and neuroactive steroid pregnanolone produced supra-additive (i.e., greater than expected effects based on the drugs alone) anxiolytic effects but infra-additive (i.e., lower than expected effects based on the drugs alone) reinforcing effects in male rhesus monkeys, suggestive of an improved therapeutic window.

**Methods:**

Female rhesus monkeys (*n*=4) self-administered triazolam, pregnanolone, and triazolam-pregnanolone combinations intravenously under a progressive-ratio schedule. In order to assess characteristic sedative-motor effects of BZ-neuroactive steroid combinations, female rhesus monkeys (n=4) were administered triazolam, pregnanolone, and triazolam-pregnanolone combinations. Trained observers, blinded to condition, scored the occurrence of species-typical and drug-induced behaviors.

**Results:**

In contrast to our previous study with males, triazolam-pregnanolone combinations had primarily supra-additive reinforcing effects in three monkeys but infra-additive reinforcing effects in one monkey. Scores for deep sedation (i.e., defined as atypical loose-limbed posture, eyes closed, does not respond to external stimuli) and observable ataxia (any slip, trip, fall, or loss of balance) were significantly increased by both triazolam and pregnanolone. When combined, triazolam-pregnanolone combinations had supra-additive effects for inducing deep sedation, whereas observable ataxia was attenuated, likely due to the occurrence of robust sedative effects.

**Discussion:**

These results suggest that significant sex differences exist in self-administration of BZ-neuroactive steroid combinations, with females likely to show enhanced sensitivity to reinforcing effects compared with males. Moreover, supra-additive sedative effects occurred for females, demonstrating a higher likelihood of this adverse effect when these drug classes are combined.

## 1. Introduction

Benzodiazepines (BZs) are positive allosteric modulators of γ-aminobutyric acid type A (GABA_A_) receptors and facilitate the modulation of chloride conductance by GABA (1–3). These drugs are among the most widely prescribed medications for the treatment of anxiety- and sleep-related disorders, but BZs have unwanted side effects including sedation and motor impairment, as well as the potential for abuse (1, 4). Considerable effort has been directed towards developing strategies to improve the therapeutic window for these drugs by augmenting the therapeutic effects and reducing or eliminating unwanted side effects.

In addition to the benzodiazepine modulatory site, GABA_A_ receptors possess a number of other distinct modulatory sites (5). One such site binds neuroactive steroids. Neuroactive steroids are endogenous (or synthetic) compounds that can function as positive allosteric modulators and can produce many of the same behavioral effects as BZs, including anxiolysis, analgesia, sedation, reinforcing and anticonvulsant effects (6–10). Importantly, neuroactive steroids can modulate a subpopulation of GABA_A_ receptors that are insensitive to classical BZs (11, 12). The similarity in effects produced by BZs and neuroactive steroids despite their different modulatory sites and distinct GABA_A_ receptor populations raises the possibility that combining a neuroactive steroid with a BZ could produce clinically-beneficial effects with lower doses of both the BZ and the neuroactive steroid. Lower doses of the component drugs presumably would produce fewer side effects.

Several groups have investigated the behavioral effects of BZ-neuroactive steroid combinations in assays of both therapeutic and abuse-related effects. In these interaction studies, combinations of BZs and neuroactive steroids engender unique profiles, although the exact nature of the interaction appears to be dependent on the drugs under study, the doses tested, and the behavioral measure. For example, using isobolographic analysis, Chuang and Reddy (13) found supra-additive (i.e., potencies of combinations greater than predicted based on the effects of the drugs alone) antiseizure effects in mice with mixtures of the BZ midazolam and the synthetic neuroactive steroids brexanolone or ganaxolone. Likewise, combinations of the BZ triazolam and the neuroactive steroid pregnanolone produced supra-additive effects in a rhesus monkey conflict model of the anxiolytic-like effects of drugs (14). Similar findings of supra-additive anxiolytic-like effects also were observed in rats with an elevated zero maze procedure (15). In contrast, infra-additive effects (i.e., potencies of combinations less than predicted based on the effects of the drugs alone) were observed for mixtures of triazolam and pregnanolone in rhesus monkeys responding under a progressive-ratio schedule of i.v. drug self-administration, an assay that measures the reinforcing effects of drugs (14). In other studies in monkeys and rats trained to discriminate midazolam or triazolam, respectively, combinations of BZs and neuroactive steroids typically produced BZ-like discriminative stimulus effects that were additive in nature [i.e., potencies of combinations were as predicted based on the effects of the drugs alone; (15, 17)]. Collectively, the results of these studies would suggest that the combination of a BZ and a neuroactive steroid does, in fact, improve the therapeutic window (i.e., supra-additive therapeutic effects with additive or infra-additive abuse-related effects).

Although the finding of infra-additive reinforcing effects of a BZ-neuroactive steroid combination in monkeys is compelling, these data were obtained with male monkeys only (14). Importantly, sex-specific behavioral and physiological effects of progesterone and progesterone-based neuroactive steroids are well documented in both human and non-human subjects (18, 19). Therefore, the present study sought to determine the extent to which infra-additive reinforcing effects of triazolam-pregnanolone combinations would be observed in a cohort of female monkeys tested under the same conditions as those used by Fischer and Rowlett (14). In addition to allowing comparisons with Fischer and Rowlett (14), triazolam and pregnanolone were chosen as pharmacological tool compounds with selectivity for BZ and neuroactive steroid sites of action on GABA_A_ receptors, and for their relatively short durations of action, which allows for interpretation of findings with fewer complications due to drug accumulation across a self-administration session.

As mentioned previously, both BZs and neuroactive steroids engender sedative-motor side effects that can limit their usage. Based on the observation that, for abuse-related side effects, combinations of BZs and neuroactive steroids engender additive or infra-additive effects, there is the possibility that these combinations will similarly engender additive or infra-additive sedative-motor effects. To assess this possibility, the effects of triazolam, pregnanolone, and triazolam-pregnanolone combinations on species-typical and drug-induced behaviors also were determined in female rhesus monkeys using an observation procedure that provides reliable metrics for drug-induced behaviors, as well as alterations of species-typical behaviors by drugs (20–24).

## 2. Materials and methods

### 2.1. Subjects and surgery

Eight adult female rhesus macaques (*Macaca mulatta*) weighing between 8 and 10 kg at the start of the study were used in the self-administration (*N* = 4) and behavioral observation (*N* = 4) procedures. Monkeys were housed individually in a colony room under a 12-h light/dark cycle (lights on at 0600 h). Monkeys had free access to water and received sufficient monkey chow to maintain healthy weights as determined by veterinary staff. Monkeys were maintained in accordance with the *Guide for Care and Use of Laboratory Animals, Eighth Edition*. Research protocols were approved by the University of Mississippi Medical Center’s Institutional Animal Care and Use Committee.

Monkeys were prepared with chronic indwelling venous catheters following the general surgical procedures described by Platt et al. (25). The external end of the catheter was fed through a fitted jacket and tether system and attached to a fluid swivel (Lomir Biomedical, Malone, NY, United States) mounted to custom-designed cage systems (Carter2 Systems, Hillsboro, OR, United States). The catheters were flushed daily with heparinized saline (100 IU/ml), and the exit site of the catheters was inspected routinely.

### 2.2. Drugs

Midazolam (Hospira Inc., Lake Forest, IL, United States) 5 mg/ml pharmaceutical stock was diluted with 0.9% saline solution. Triazolam (Sigma-Aldrich, St. Louis, MO, United States) was dissolved in propylene glycol and diluted with sterile water to a 50% propylene glycol/50% sterile water solution. Pregnanolone (Tocris Bioscience, Bristol, United Kingdom) was dissolved in a 45% (w/v) 2-hydroxypropyl-β-cyclodextrin solution. Triazolam-pregnanolone combinations for the self-administration study were prepared by dissolving each drug separately at twice the concentration of the test dose. The separate solutions were then combined in a single syringe prior to test sessions to create the test combination. Triazolam-pregnanolone combinations for the observation study were prepared by dissolving each drug separately and then administered sequentially via the i.v. catheter. In these sessions, the test dose of triazolam was administered first, followed by the test dose of pregnanolone.

### 2.3. Self-administration procedure

Using the procedure described by Fischer and Rowlett (14), four female rhesus monkeys were trained to self-administer the BZ midazolam (0.056 mg/kg/injection) under a progressive-ratio (PR) schedule of i.v. drug injection. At the beginning of a daily session, a set of two white stimulus lights above a response lever was illuminated. Upon completion of a response requirement, the white lights were extinguished and a set of two red lights was illuminated for 1-s, coinciding with an injection. Each trial ended with either an injection or the expiration of a 30-min limited hold. Trials were separated by a 30-min timeout period, during which all lights were off and responding had no programmed consequences.

Daily experimental sessions consisted of five components made up of four trials each. The response requirement remained constant for each of the four trials within a component, but doubled during each subsequent component. The session ended when a monkey self-administered a maximum of 20 injections or when the response requirement was not completed for two consecutive trials. The PR schedule for three monkeys (identification numbers = 318-01, 143-03, and 388-06) consisted of a sequence of response requirements: 40, 80, 160, 320, and 640 responses per injection. The schedule for the fourth monkey (165-01) consisted of response requirements: 20, 40, 80, 160, and 320 responses per injection.

Once training was complete, midazolam or saline was made available on alternating baseline days and until responding was stable (i.e., ≥10 injections on midazolam sessions and ≤5 injections on saline sessions). Test (T) sessions with triazolam, pregnanolone, or triazolam-pregnanolone combinations were added to the alternating sequence of midazolam (M) and saline (S) sessions according to the following sequence: MTSMTSTMST, etc. The ratios of triazolam-pregnanolone used in test combinations were calculated from the ED_50_ values of triazolam and pregnanolone dose-response curves for each monkey (see Section 2.5.1 for description of ED_50_ determinations). From these values, triazolam-pregnanolone combinations of individualized relative potencies (26) of 1:0.3, 1:1, 1:3 were tested (see Supplementary Table S3 for actual dose combinations tested). Each dose/dose combination was evaluated at least twice. The individual triazolam:pregnanolone dose ratios varied considerably; therefore individual subject’s data are shown for the self-administration studies. The individual ED_50_ values and dose ratios used to determine each combination are listed in Table 1.

**Table 1:** Individual ED_50_ and triazolam:pregnanolone dose ratios used in self-administration.

	ED_50_ (mg/kg)		Triazolam: pregnanolone relative potency dose ratios		
Monkey	Triazolam	Pregnanolone	1:0.3	1:1	1:3
143-03	0.0040	0.064	1:5	1:16	1:48
318-01	0.0006	0.020	1:11	1:33	1:100
165-01	0.0011	0.040	1:12	1:36	1:109
388-06	0.0015	0.073	1:16	1:49	1:146

### 2.4. Behavioral observation procedure

Behavioral observations were conducted using the focal animal sampling approach as described in Platt et al. (27) and modified for rhesus monkeys [cf., (20, 21, 28, 29)]. Observers (four total) met a 90% inter-observer reliability criterion prior to the experiments and were blind to the drug treatments. Twenty-six species-typical and characteristic drug-induced behaviors (see Supplementary Table S1 for all definitions of behaviors) were scored by recording each instance that a particular behavior occurred during 15-s intervals in a 5-min observation period.

For sedation measures, structured exposure to stimuli were included in the observation sessions (20). When a monkey was observed to have closed eyes, an assessment of the animal’s responsiveness to the stimuli was determined. Specifically, the observer presented three stimuli: (1) walked at a normal pace towards the cage, (2) spoke the animal’s name, and (3) tapped twice on the cage bars or moved the lock used to secure the door of the cage. If the monkey responded immediately (i.e., opened eyes and oriented to the observer), *rest/sleep posture* was scored. If the monkey attended more slowly (i.e., >3 s following stimuli) and was observed to be assuming an atypical posture that differed from the characteristic rest/sleep posture (e.g., unable to keep an upright posture), the observer scored *moderate sedation*. If the monkey did not open eyes across the 15-s interval after all three stimuli, the observer noted the loss of ability to respond to external stimuli and scored *deep sedation*. The assessment of sedation was initiated during the 5-min sampling period if the animal presented, at any time during that period, with its eyes closed. The result of this assessment was recorded for each remaining 15-s interval of the 60-s epoch unless eyes opened. Afterwards, eyes closing again reinitiated the assessment. If eyes remained closed, then the assessment was repeated at the beginning of the next 60-s epoch.

Monkeys were habituated to the observers’ presence over several weeks prior to testing. Baseline data were collected following saline injections. The effects of triazolam and pregnanolone alone were determined first, followed by combinations of triazolam and pregnanolone. The ratios of triazolam-pregnanolone were based on 1:1, 1:3, and 1:9 proportions, in order to focus on primarily sedative-motor effects that occurred at the higher dose ranges of these drugs (see Supplementary Table S4 for actual dose combinations tested). Scoring occurred at 5, 10, 20, 40, 80, and 160 min after the i.v. injection. Different doses of each drug or drug combination were evaluated in a randomized order, with at least a 2-day drug-free period between tests. Unlike the self-administration data, in which variance in potencies was relatively high among the monkeys, the low variability in observation allowed for use of group statistics.

### 2.5. Data analysis

All statistical tests were conducted using GraphPad Prism Version 8.4.3 for Windows (GraphPad Software, La Jolla, CA, United States). Parametric statistics were used unless noted otherwise, and for all analyses involving multiple conditions, the error rate (α) was constrained to *p* ≤ 0.05.

#### 2.5.1. Self-administration

Data for self-administration consisted of the number of injections/session as well as the last response requirement completed (break point, BP). For triazolam and pregnanolone alone, self-administration was analyzed initially by separate one-way repeated measures analysis of variance (ANOVA) and Bonferroni tests comparing each dose vs. vehicle tests. BP data were used to calculate BP_max_, which is the maximum BP obtained for individual monkeys for a test drug, irrespective of dose. This measure was used to compare the relative reinforcing effectiveness of each test drug, as well as combinations vs. the drugs alone. Because of violations in homogeneity of variance, BP_max_ data were analyzed with non-parametric Friedman’s ANOVA with Dunn’s multiple comparison tests. To determine potencies, ED_50_ values for each test drug were determined by analyzing the data points encapsulated by the peak and trough on the ascending limb and conducting log-linear regression, or linear interpolation when only two data points were available. The drug combination data were analyzed primarily with isobolographic and dose addition methods, described in Section 2.5.3.

#### 2.5.2. Behavioral observation

For each subject, scores for each behavior were calculated as the number of 15-s intervals in which the behavior occurred (max score = 20 in a 5-min observation period). These scores were averaged across subjects to obtain a group mean for each dose of each drug at each time point. Drug effects on each behavior were evaluated by conducting a two-way analysis of variance (ANOVA) with both time and dose as within-subject factors. Bonferroni’s multiple-comparison tests were conducted to compare the effects of each dose of triazolam, pregnanolone, and triazolam-pregnanolone combinations to vehicle controls. Dose-response functions additionally were constructed by computing cumulative scores over the entire time period (20), which were used to calculate ED_50_ values using the same approach described for self-administration (log-linear regression or linear interpolation). The primary method for analyzing combined effects of the two drugs was isobolographic and dose-addition analyses, described in the next section.

#### 2.5.3. Isobolographic and dose-addition analyses

Two methods of analyses were used to evaluate drug combination effects. First, the effects of triazolam-pregnanolone combinations were assessed graphically with the use of isobolograms (26, 30). Isobolograms were constructed by connecting the ED_50_ of triazolam alone plotted on the y axis with the ED_50_ of pregnanolone plotted on the x axis. The line of additivity connects these points and contains the loci of dose combinations that would produce an ED_50_ equal to the ED_50_ of pregnanolone or triazolam administered alone if the combination is additive. Dose combinations that fall below or to the left of the line of additivity indicate an ED_50_ was reached with lesser quantities of the drugs, suggestive of supra-additivity. In contrast, dose combinations that fall above or to the right of the line of additivity are suggestive of infra-additivity. Theoretical additive dose combinations (*a*, *b*) are described by the equation (30):

(1) $$B=b+\frac{B_{50}}{{\left[\frac{E_{B}}{E_{c}\left(1+\frac{A_{50}^{q}}{a^{q}}\right)}-1\right]}^{\frac{1}{p}}}$$

This equation adjusts for differences in maximum effect, which in the present study was manifest as pregnanolone having a lower maximal effect than triazolam for some monkeys. A given dose of pregnanolone was designated as *A* with *B* as a given dose of triazolam. The potencies (ED_50_ values) of pregnanolone and triazolam when administered alone were defined as *A*_50_ and *B*_50_, respectively. The maximum effects of triazolam and pregnanolone were defined as *E_B_* and *E_c_*, respectively. The coefficients *p* and *q* refer to curve-fitting parameters (i.e., Hill coefficients).

Drug combination effects also were analyzed by comparing the experimentally determined ED_50_ values for each mixture (*Z*_mix_) with predicted additive ED_50_ values (*Z*_add_) as described by Tallarida (31). *Z*_mix_ was defined as the total drug dose (i.e., dose triazolam + dose pregnanolone) that produced an increase to 50% of the maximum effect in drug self-administration or observable behavioral effect. Across all endpoints, the mean experimentally determined ED_50_ values (*Z*_mix_) and predicted additive ED_50_ values (*Z*_add_) for each mixture were compared with a paired *t*-test. An interaction index (*γ*) also was calculated to quantify deviation from additivity for each drug combination (32). From this calculation, a *γ* value of 1 indicated additivity, *γ* values that approached 0 indicated a greater degree of supra-additivity, and *γ* values greater than 1 indicated a greater degree of infra-additivity. One-sample *t*-tests were calculated to determine if the mean of the interaction indexes for each combination were significantly different from the theoretical additive value of 1.

## 3. Results

### 3.1. Drug self-administration

#### 3.1.1. Drugs alone

**Figure 1:**
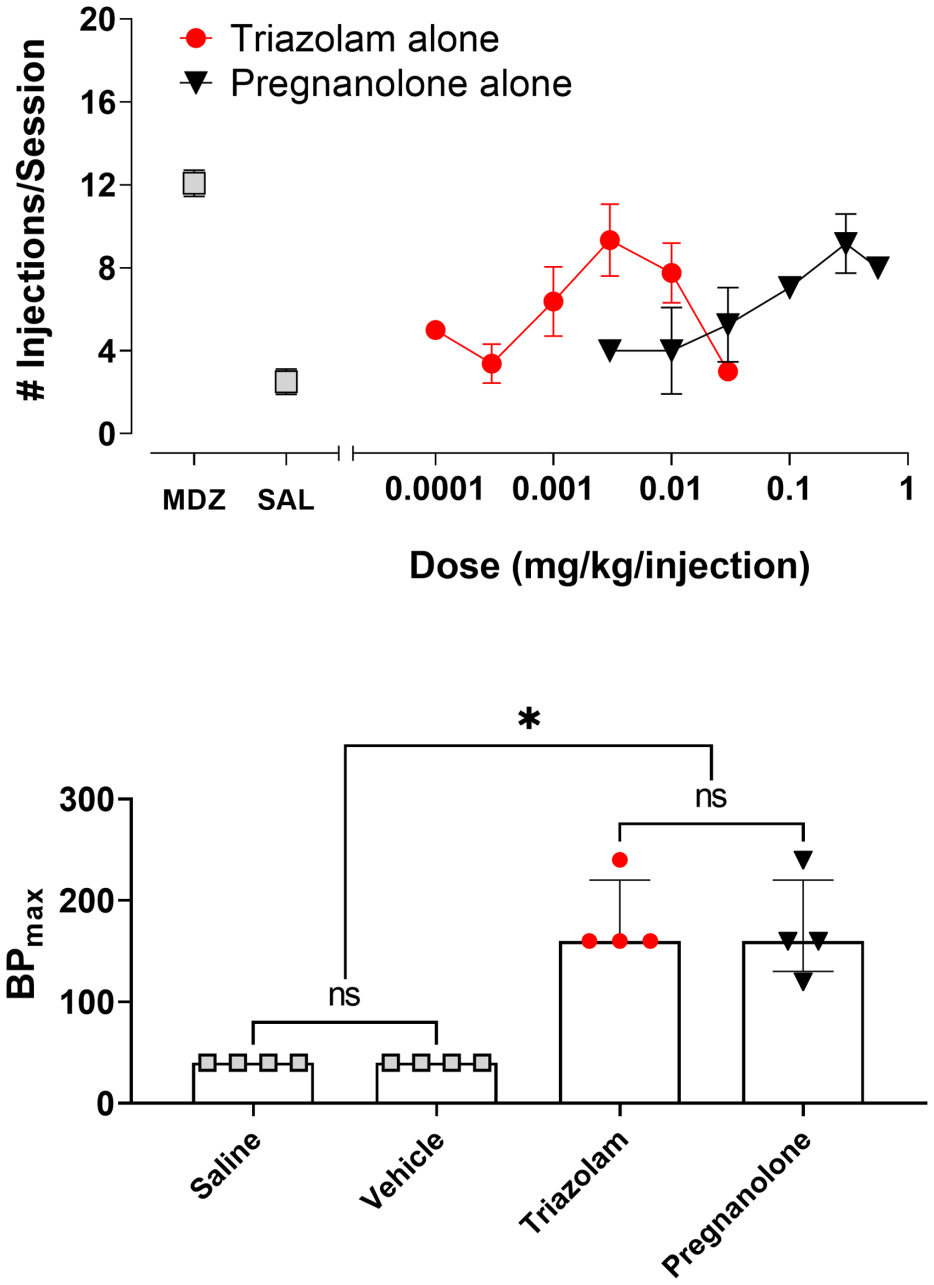
Self-administration of triazolam and pregnanolone under the progressive-ratio schedule of self-administration (*N* = 4 female rhesus monkeys). Top panel: Mean number of injections/session ± SEM for baseline self-administration of midazolam (point above “MDZ”), saline (point above “SAL”), and a range of doses of triazolam and pregnanolone alone. Bottom panel: Relative reinforcing effectiveness of saline, vehicle, triazolam and pregnanolone alone. Data are median BPmax (highest breakpoint achieved, irrespective of dose, with breakpoint defined as the last response requirement completed in a progressive-ratio response sequence) and inter-quartile ranges. Note that *p < 0.05 for both saline and vehicle compared with triazolam and pregnanolone, and “ns” indicates lack of significance between saline and vehicle, as well as triazolam and pregnanolone (Dunn’s multiple comparison tests).

Figure 1 shows the reinforcing effects of triazolam and pregnanolone alone under the PR schedule of drug self-administration. Also shown are baseline values for midazolam and saline self-administration (Figure 1, top panel). On baseline days, the training dose of midazolam maintained 12.1 ± 0.6 injections/session and saline maintained 2.5 ± 0.6 injections/session. Vehicle levels (3.0 ± 0.5 injections/session, not shown on graph) were not different from saline levels. As can be seen in the top panel of Figure 1, triazolam availability resulted in an increase in the mean number of injections/session relative to saline/vehicle levels up to 0.003 mg/kg/injection, followed by a decrease at higher doses, i.e., an inverted U-shaped function [repeated measures ANOVA: *F* (4, 12) = 7.40, *p* = 0.026 for vehicle and all triazolam doses]. No individual dose resulted in mean number of injections/session that differed from vehicle (Bonferroni *t*-tests, *p*’s > 0.05). Similarly, pregnanolone availability resulted in an increase in the mean number of injections/session, although no appreciable decreases in self-administration were evident at doses up to 0.56 mg/kg/injection [repeated measures ANOVA: *F* (4, 12) = 11.73, *p* = 0.050 for vehicle and all pregnanolone doses]. Bonferroni tests showed that both 0.3 and 0.56 mg/kg/injection maintained higher injections/session than vehicle (*p*’s < 0.05).

To compare relative reinforcing effectiveness among the two drugs, saline, and vehicle, the median BP_max_ values with interquartile ranges are shown in the bottom panel of Figure 1. Friedman’s ANOVA showed a significant overall effect for all conditions: *Q* = 11.06, *p* = 0.0046. Dunn’s multiple comparison’s tests revealed that although both saline and vehicle values were significantly different from BP_max_ values for triazolam and pregnanolone, no statistically significant differences existed for either saline vs. vehicle or triazolam vs. pregnanolone.

#### 3.1.2. Drug combinations

**Figure 2:**
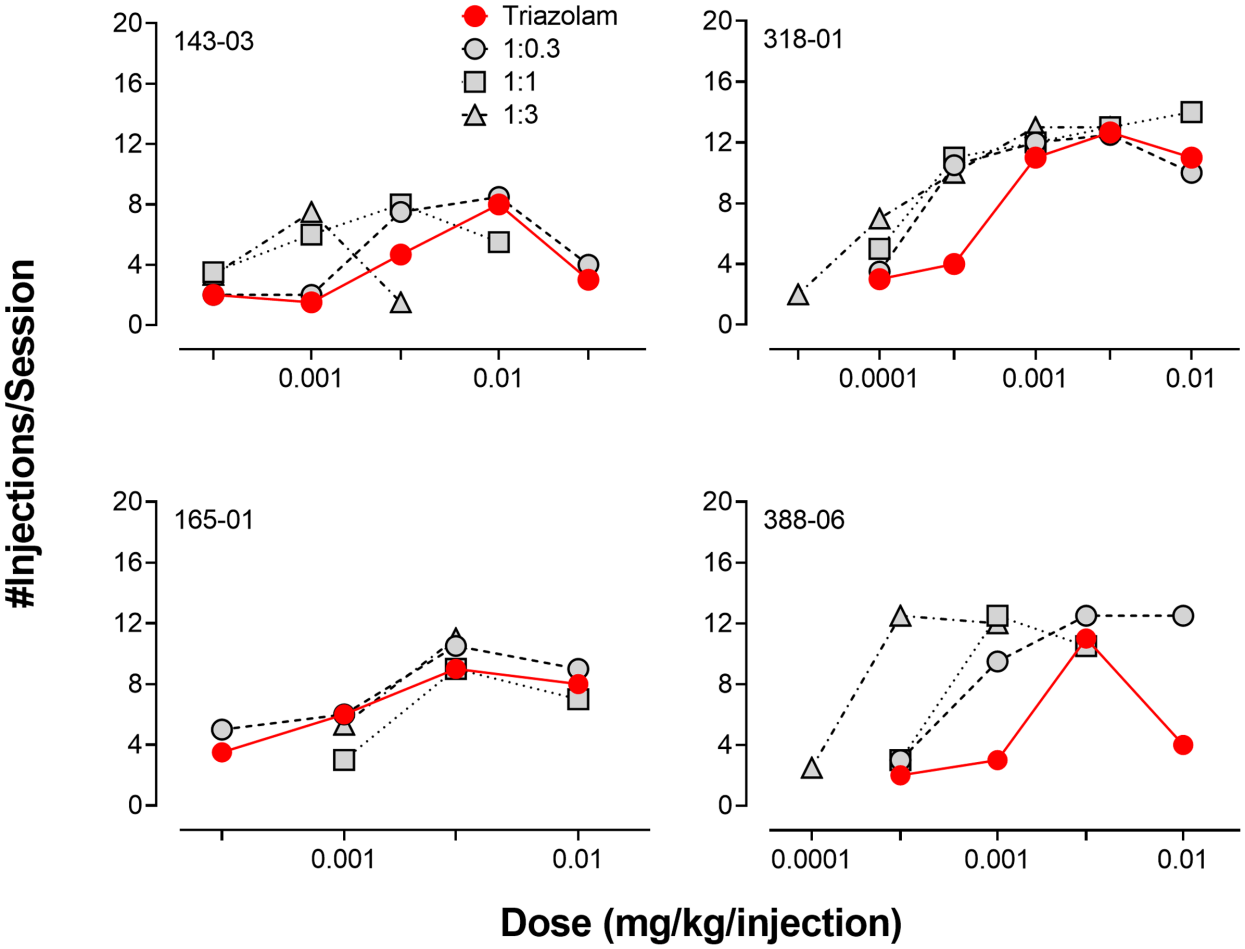
Comparison of self-administration of triazolam vs. triazolam-pregnanolone combinations in female rhesus monkeys responding under a progressive-ratio schedule (*N* = 4 monkeys). Each individual panel depicts data for an individual monkey, identified by the 5-digit code in the upper left hand part of the panel. Ratios are fixed proportions of triazolam to pregnanolone, with three ratios tested: 1.0:0.3, 1.0:1.0, 1:0:3.0. Data are mean number of injections/session.

**Figure 3:**
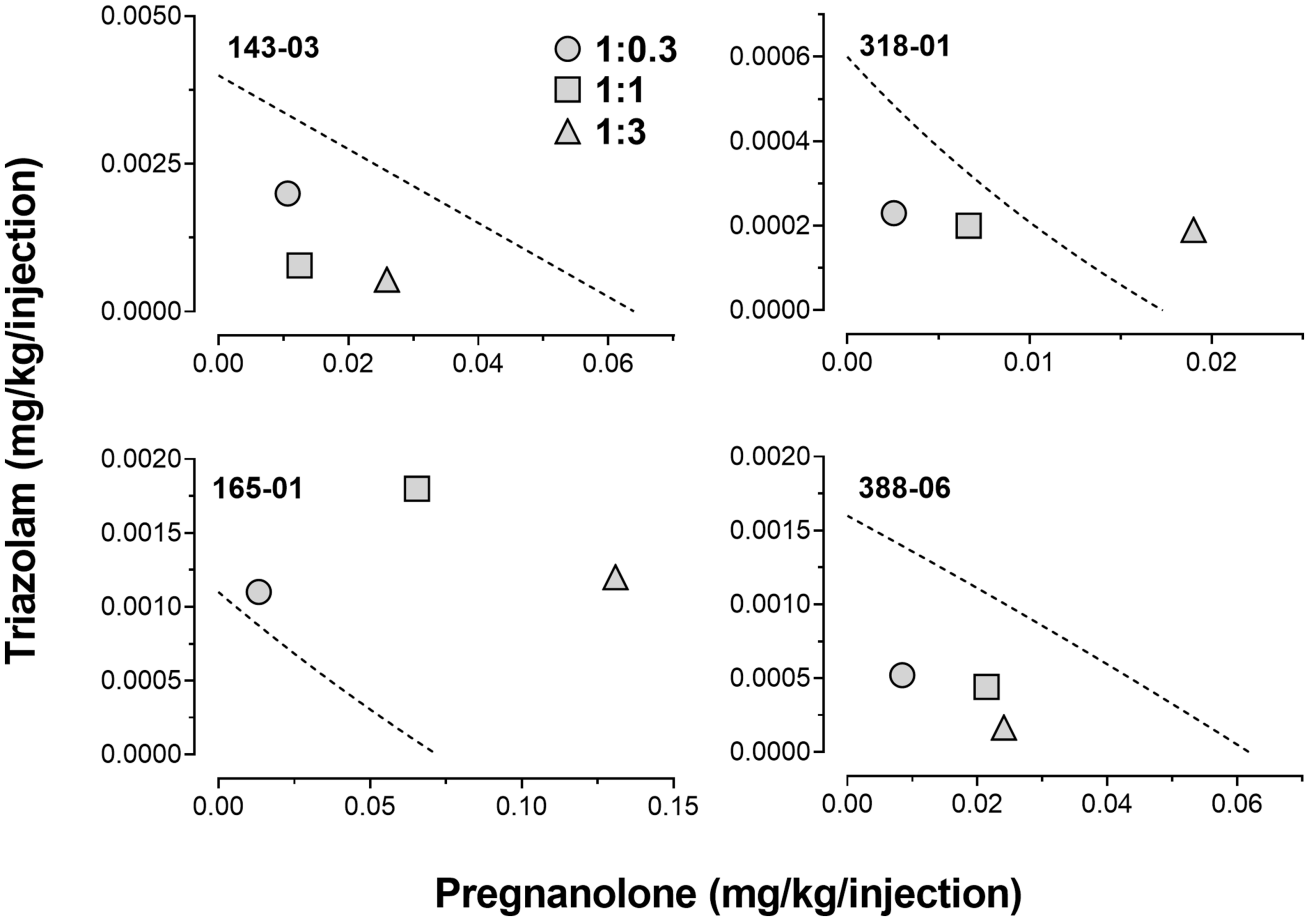
Isobolograms for triazolam-pregnanolone combinations in the progressive-ratio self-administration procedure. Each individual panel depicts data for an individual monkey, identified by the 5-digit code in the upper left hand part of the panel. Dashed lines represent theoretical lines of additive combinations for triazolam and pregnanolone (calculated via Equation 1, see text). The ED50 values for triazolam are plotted on the y-axes as a function of the corresponding ED50 values for pregnanolone on the x-axes. Individual data points represent ED50 values for combined effects of the two drugs, in proportions of 1:0.3, 1.0:1.0, and 1.0:3.0 triazolam to pregnanolone. Values below the line of additivity represent supra-additive interactions, whereas values above the line of additivity represent infra-additive interactions. Points close to or on the additivity line represent additive effects (i.e., no interaction).

**Figure 4:**
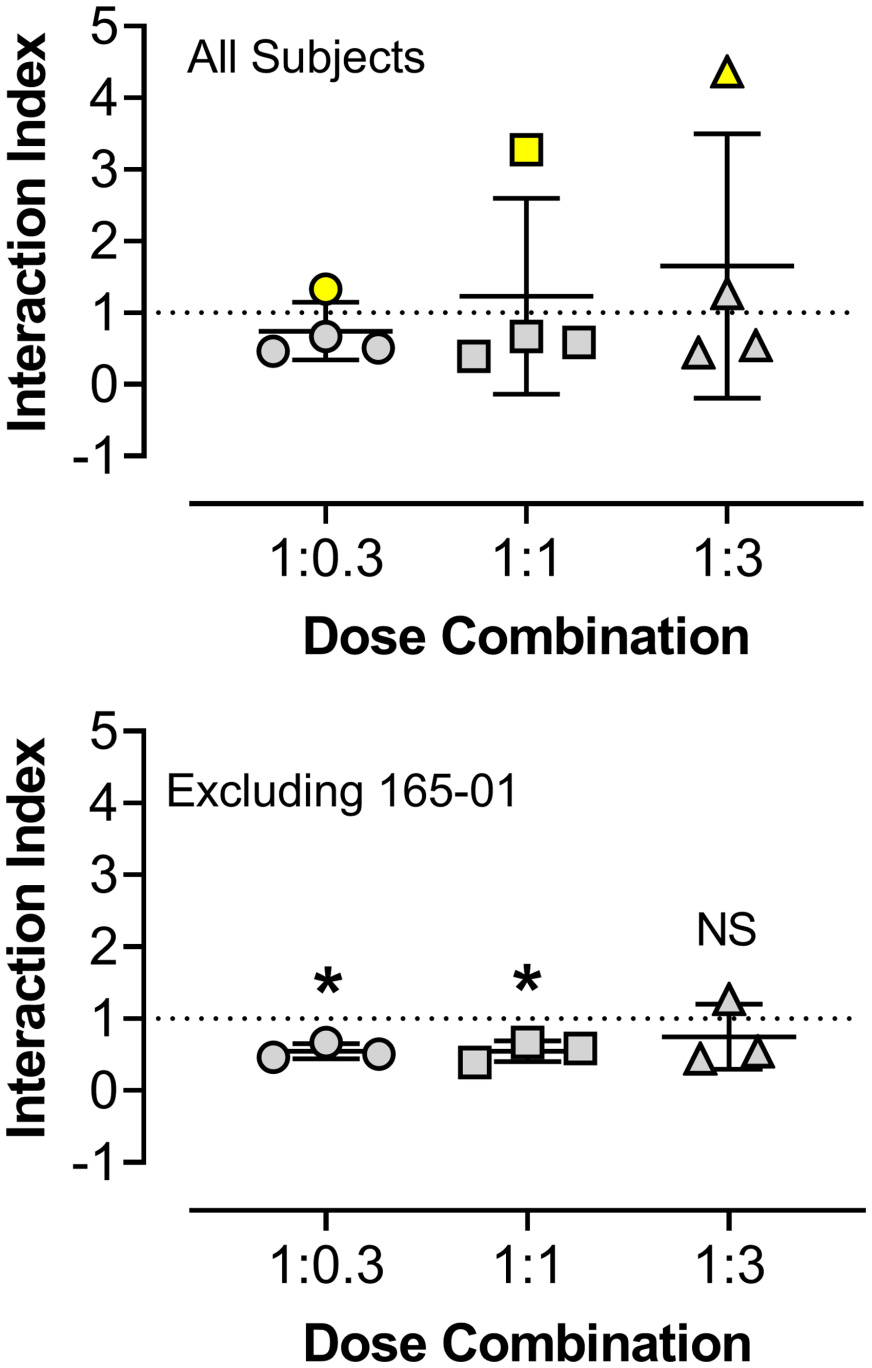
Statistical analyses of γ interaction index values obtained from dose-addition analyses of the combined reinforcing effects of triazolam and pregnanolone under the progressive-ratio procedure. The y-axis shows the interaction index values and the x-axis depicts the ratios of triazolam to pregnanolone. Symbols represent the four female rhesus monkeys. The top panel shows data for all subjects, with no significant differences observed vs. the theoretical value of 1.0 (additivity, one-sample *t*-tests, *p*’s > 0.05). The bottom panel shows the analysis when subject 165-01 (yellow symbols in the top panel) was omitted from the statistical tests. For the 1:0.3 and 1:1 ratios of triazolam-pregnanolone, the interaction indices were significantly different from 1.0 (*p < 0.05, one sample *t*-tests), whereas the 1:3 ratio did not differ from 1.0, i.e., additive effects were observed at this ratio combination.

Due to the relatively large degree of variability among animals in the three drug combination conditions, individual subjects’ data are presented in four separate panels for clarity (Figure 2). For two monkeys (143-03 and 388-06), combining triazolam and pregnanolone resulted in a proportion-dependent shift to the left in the triazolam dose-response function. For monkey 318-01, all ratios shifted the triazolam dose-response function to the left to essentially the same degree, whereas for monkey 165-01, no clear shift in the triazolam dose-response function was evident (the 1:1 ratio engendered a moderate rightward shift). Figure 3 shows the corresponding isobolograms based on the ED_50_ values of the drugs alone and combined, with dashed lines representing the theoretical lines of additivity derived from Equation (1). Note that two of the lines of additivity have slightly concave shapes, indicative of differences in the maximum number of injections/session obtained for triazolam vs. pregnanolone for these monkeys (318-01 and 165-01). Three of the four monkeys demonstrated 2–3 ratios that were below the line of additivity, suggesting supra-additive interactions for the reinforcing effects of triazolam and pregnanolone combined. Strikingly, monkey 165-01 showed additive effects (1:0.3) and infra-additive interactions (1:1 and 1:3), effects that clearly were in the opposite direction of the other monkeys. Examination of the dose-addition analysis for these combinations (Table 2) corroborated the overall results from the isobolographic analyses, with γ interaction indices showing values less than 1.0 for all ratios in three of the four monkeys, and *γ* values greater than 1.0 for the ratios in monkey 165-01. Statistical analyses of the γ interaction index data are shown in Figure 4, with data in the top panel including all subjects and data in the bottom panel excluding monkey 165-01. One sample *t*-tests conducted on the data in the top panel showed no significant differences from the theoretical value of 1.0; however, when 165-01 was excluded, the interaction indices for the 1:0.3 and 1:1 ratios, but not 1:3 ratio, were significantly below 1.0 (*p*’s < 0.05). Therefore, combinations of triazolam and pregnanolone in ratios of 1:0.3 and 1:1 had supra-additive reinforcing effects. That is, lower combined doses resulted in reinforcing effects than would be expected if the reinforcing effects were simply additive when combined.

**Table 2:** Predicted additive potency (*Z*_add_) and experimentally determined potency (*Z*_mix_) of triazolam-pregnanolone combinations on self-administration.

	Triazolam-pregnanolone combination								
	1:0.3			1:1			1:3		
Monkey	Z_add_	*Z*_mix_	γ*	*Z*_add_	*Z*_mix_	γ*	*Z*_add_	*Z*_mix_	γ*
143-03	0.019	0.013	0.67	0.034	0.013	0.39	0.049	0.027	0.56
318-01	0.0055	0.0028	0.51	0.010	0.0069	0.67	0.015	0.019	1.27
165-01	0.011	0.014	1.33	0.021	0.067	3.27	0.030	0.13	4.36
388-06	0.019	0.0090	0.46	0.037	0.022	0.59	0.055	0.024	0.56

* γ interaction index (i.e., *Z*_mix_/*Z*_add_).

In addition to her data being strikingly different from the other monkeys, 165-01 was excluded for the analysis of interaction indices after a review of records of the subject’s health revealed that over the course of ~6 months, this monkey showed no observable menses. This differed from the other subjects which all showed regular menses occurring approximately every month. Clinical hormonal assays using mass spectroscopy conducted with plasma samples from 165-01 revealed undetectable levels of progesterone, which combined with the lack of menses and the monkey’s age (20 years at the time of the study) resulted in a clinical diagnosis of menopause, albeit at an age younger than is typical for rhesus monkeys.

Finally, BP_max_ values were analyzed for all combinations (and all monkeys) and no statistically significant effects were obtained when these values for the three ratios were included with the BP_max_ values for the drug alone. This included analyses that excluded monkey 165-01 (Friedman’s ANOVA and Dunn’s tests for all possible pairwise comparisons, *p*’s > 0.05; data not shown).

### 3.2. Behavioral observation

#### 3.2.1. Summary of behavioral effects

Under baseline and vehicle conditions for triazolam and pregnanolone testing, monkeys displayed varying degrees of species-typical behavior, with self-groom, passive visual, tactile/oral exploration, forage, scratch, and locomotion being the most common behaviors (data not shown). Little to no behaviors indicative of sedative effects (e.g., rest/sleep posture or moderate/deep sedation) were observed during baseline conditions and vehicle sessions.

Of the 26 behaviors quantified during testing with triazolam, pregnanolone, and the three combinations (1:1, 1:3, 1:9), the majority of species-typical behaviors were not altered significantly (Supplementary Table S2). Exceptions were decreases in forage for triazolam alone, scratch and groom for the 1:3 combination, and groom for the 1:9 combination (Supplementary Table S2). In contrast, the two drugs alone and the combinations consistently increased measures of observable ataxia and deep sedation (note that rest/sleep posture and moderate sedation were not altered by any test condition). Because of the consistent and statistically significant effects on observable ataxia and deep sedation, these measures were chosen for isobolographic and dose-addition analyses, as described below.

#### 3.2.2. Deep sedation

**Figure 5:**
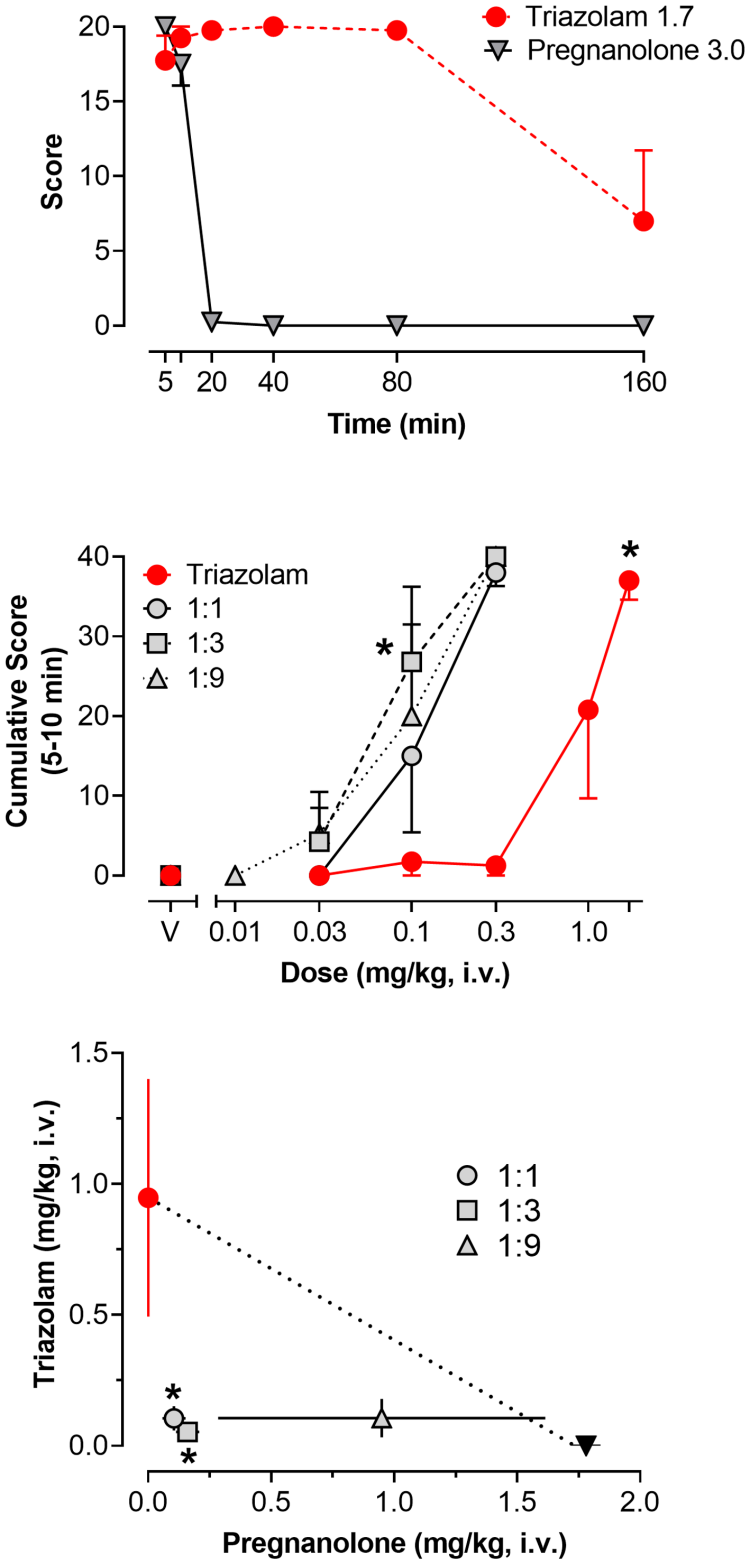
Deep sedation, scored *via* quantitative observation, following i.v. administration of triazolam and pregnanolone, alone and combined, in female rhesus monkeys (*N* = 4). Top panel: Mean modified frequency scores ± SEM as a function of time for deep sedation at doses significantly different from vehicle for triazolam and pregnanolone (1.7 mg/kg and 3.0 mg/kg, respectively). Middle panel: Dose-response functions for deep sedation after triazolam and triazolam-pregnanolone combinations (fixed ratio proportions of 1:1, 1:3, 1:9). Data are mean cumulative score ± SEM for scores cumulated during the 5–10 min observation sessions. Note that *p < 0.05 vs. vehicle (V), Bonferroni *t*-tests. Bottom panel: Isobologram for the data depicted in the middle panel. Dashed lines represent theoretical lines of additive combinations for triazolam and pregnanolone (calculated via Equation 1, see text). The ED50 values for triazolam are plotted on the y-axes as a function of the corresponding ED50 values for pregnanolone on the x-axes. Individual data points represent ED50 values for combined effects of the two drugs, in proportions of 1:0.3, 1.0:1.0, and 1.0:3.0 triazolam to pregnanolone. Values below the line of additivity represent supra-additive interactions, whereas values above the line of additivity represent infra-additive interactions. Points close to or on the additivity line represent additive effects (i.e., no interaction). Error bars represent SEM values, and note that *p < 0.05 based on dose addition analyses.

Significant increases in deep sedation were detected as a function of dose and time for both triazolam and pregnanolone alone [2-within repeated measures ANOVAs; triazolam dose × time interaction, *F*(25, 75) = 1.70, *p* = 0.042; pregnanolone dose × time interaction, *F*(20, 60) = 272.8, *p* < 0.0001]. For clarity, the top panel of Figure 5 shows the effects of the highest doses tested for triazolam (1.7 mg/kg, i.v.) and pregnanolone (3.0 mg/kg, i.v.). A striking difference between the two drugs is that triazolam engendered deep sedation from 5 to 80 min post-injection, whereas pregnanolone engendered deep sedation from 5 to 20 min post-injection only (Bonferroni multiple comparisons, *p*’s < 0.05). Because of this difference in time course, analysis of the effects of the drug combinations were limited to the 5–10 min data only (i.e., scores were cumulated from 5 to 10 min). The middle panel of Figure 5 shows the mean cumulative scores for triazolam and the 1:1, 1:3, and 1:9 ratios of triazolam to pregnanolone as a function of dose. As can be seen in the Figure 5, all three ratios resulted in a leftward shift in the triazolam dose-response function, consistent with an overall enhanced effect on deep sedation by this drug combination.

Figure 5 (bottom panel) and Table 3 show the results of isobolographic and dose-addition analyses of the deep sedation data with triazolam and pregnanolone. As can be seen in the Figure 5, the 1:1 and 1:3 combinations were clearly below the theoretical line of additivity for this behavioral measure. The mean ED_50_ for the 1:9 combination also was below the line of additivity; however, the error bars associated with the pregnanolone effects clearly overlapped the additivity line. These observations were confirmed with the dose-addition analysis (Table 3), in which the experimentally determined ED_50_ values (*Z*_mix_) for the 1:1 and 1:3 combinations were significantly less than the predicted additive ED_50_ values (*Z*_add_; [1:1] *t*(3) = 6.38, *p* < 0.01; [1:3] *t*(3) = 8.50, *p* < 0.01), but no such significant difference was detected for the 1:9 combination. Similarly, one-sample *t*-tests showed that the γ interaction indices for the 1:1 and 1:3, but not 1:9 combinations, were significantly lower than the theoretical value of 1.0 (*p*’s < 0.05). In summary, these results indicate that combinations of triazolam and pregnanolone in ratios of 1:1 and 1:3 but not 1:9 had a supra-additive effect in inducing deep sedation. That is, combinations of lower doses of the component drugs resulted in significant levels of deep sedation that would not be expected if the drug effects were simply additive.

**Table 3:** Predicted additive potency (*Z*_add_) and experimentally determined potency (*Z*_mix_) of triazolam-pregnanolone combinations on deep sedation.

Drug combination	*Z*_add_ (±95% CI)	*Z*_mix_ (±95% CI)	γ^†^
1:1	1.18 (0.39)	0.21 (0.09)*	0.18
1:3	1.39 (0.28)	0.21 (0.06)*	0.15
1:9	1.59 (0.15)	1.06 (0.74)	0.66

* An experimentally determined potency significantly different from the predicted additive potency (*p* < 0.05).

† γ interaction index (i.e., *Z*_mix_/*Z*_add_).

#### 3.2.3. Observable ataxia

**Figure 6:**
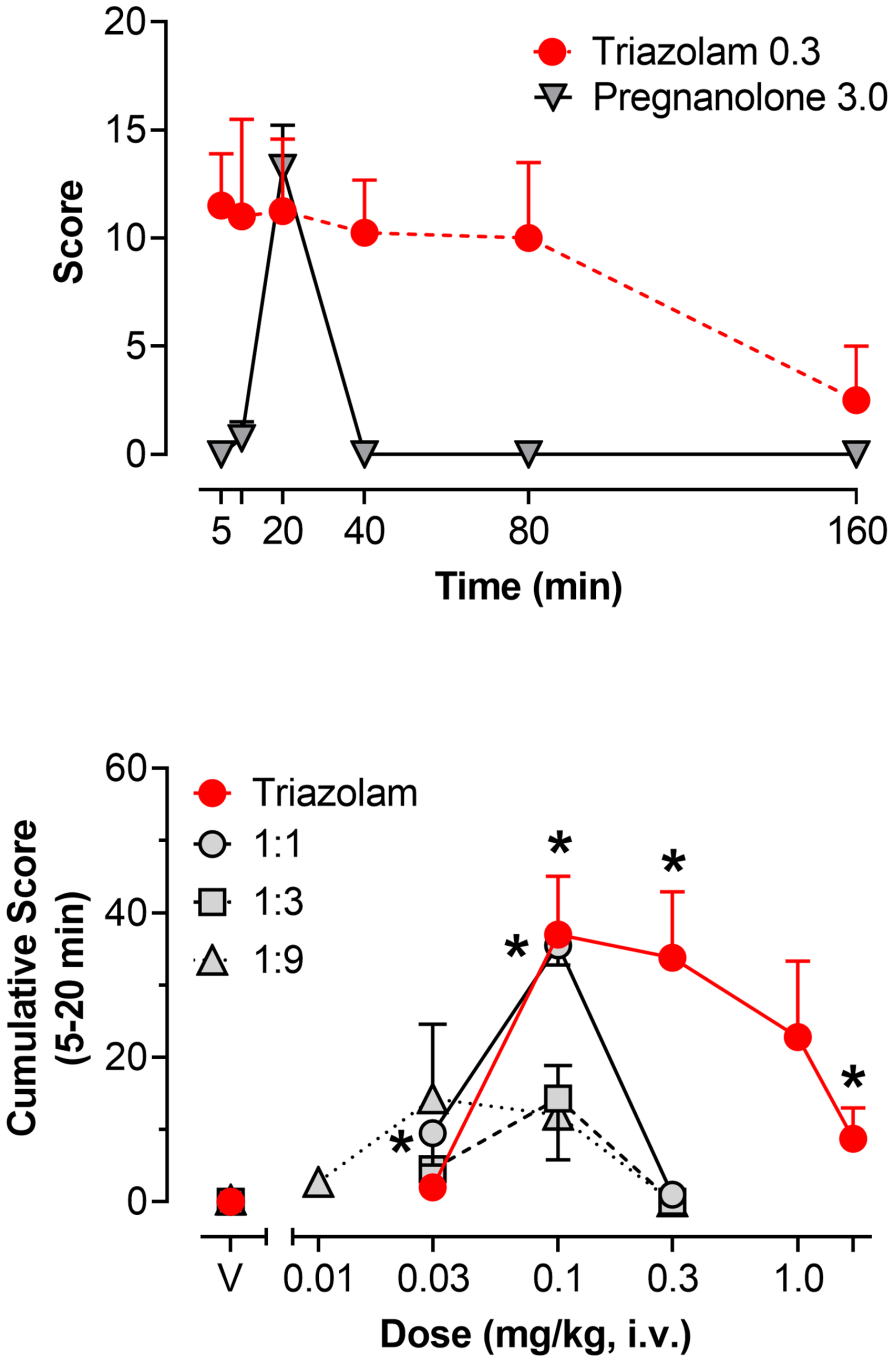
Observable ataxia, scored *via* quantitative observation, following i.v. administration of triazolam and pregnanolone, alone and combined, in female rhesus monkeys (*N* = 4). Top panel: Mean modified frequency scores ± SEM as a function of time for deep sedation at doses significantly different from vehicle for triazolam and pregnanolone (0.3 mg/kg and 3.0 mg/kg, respectively). Bottom panel: Dose-response functions for deep sedation after triazolam and triazolam-pregnanolone combinations (fixed ratio proportions of 1:1, 1:3, 1:9). Data are mean cumulative score ± SEM for scores cumulated during the 5–20 min observation sessions. Note that *p < 0.05 vs. respective vehicle (V), Bonferroni *t*-tests.

The pattern of effects observed for observable ataxia for the two drugs was somewhat more complex than that for deep sedation. In this regard, no dose × time interaction was evident for triazolam alone, although the main effect of dose was significant [*F*(5, 15) = 5.048, *p* = 0.0065]. Bonferroni *t*-tests revealed that the 0.3 mg/kg dose of triazolam showed an overall increase in the mean score for observable ataxia, irrespective of time (*p* < 0.05), and this dose is plotted in the top panel of Figure 6. Pregnanolone, in contrast, did show a significant dose × time interaction [*F*(20, 60) = 33.97, *p* < 0.0001]. The highest dose of pregnanolone is shown in Figure 6, and as with deep sedation, a relatively transient effect on observable ataxia was observed (significant increase above vehicle levels at the 20-min time point only, Bonferroni *t*-test, *p* < 0.05). Therefore, for observable ataxia, the dose combination analyses were conducted for the 5–20 min time points only.

Figure 6 (bottom panel) shows the dose-response functions for triazolam and the 1:1, 1:3, and 1:9 combinations of triazolam-pregnanolone as cumulative scores for the 5–20 min time period. Triazolam alone increased mean cumulative scores for observable ataxia up to 0.1 mg/kg; however, this effect dissipated as the dose of triazolam was increased, i.e., an inverted U-shaped function was observed (Bonferroni *t*-tests, *p*’s < 0.05). The lowest ratio, 1:1, appeared to result in a leftward shift of the descending limb of the triazolam dose-response function, but not the ascending limb. For the two higher ratios, the dose-response function appeared to be shifted primarily downward, with no significant effects on observable ataxia obtained by either ANOVA or Bonferroni tests. Because these dose-response functions were essentially flat, no ED_50_ values could be computed and therefore isobolographic and dose-addition analyses were not conducted. Although these overall effects may be interpreted as an attenuation of the effects of triazolam on observable ataxia, it is important to note that the dose range of 0.01–0.3 mg/kg of triazolam was also where significant supra-additive effects on deep sedation occurred, raising the likely possibility that deep sedation masked the occurrence of observable ataxia.

## 4. Discussion

The present study evaluated the extent to which combinations of the BZ, triazolam, and the neuroactive steroid, pregnanolone, resulted in significant changes in reinforcing and sedative-motor effects in female rhesus monkeys. Regarding reinforcing effects, we previously found that self-administration of triazolam-pregnanolone combinations resulted in infra-additive interactions or additive effects in male monkeys (14). In the present study, we used the PR self-administration methods of Fischer and Rowlett (14) to repeat the combination studies in a group of female rhesus monkeys. Although the fact that these studies were not direct comparisons (i.e., females and males in a single experiment), there were apparent sex differences in self-administration, both for the drugs alone as well as combined. As shown in Table 4, triazolam and pregnanolone were 4- to 2-fold less potent in females compared with males, respectively, and showed 15–34% lower BP_max_ values, respectively, suggesting that both drugs were moderately less potent and effective as reinforcers in females vs. males. More strikingly, however, was the pattern of interactive effects between the two studies. In this regard, while the male monkeys in Fischer and Rowlett (14) demonstrated infra-additive to additive reinforcing effects when triazolam and pregnanolone were combined, three of four female monkeys in the present study showed robust supra-additive effects. The fourth monkey demonstrated infra-additive to additive effects; however, this monkey clinically presented with signs of menopause, including evidence of relatively low circulating progesterone levels. Based on these results, we propose that progesterone-derived neuroactive steroids (e.g., allopregnanolone) may act to enhance the reinforcing effects of triazolam and pregnanolone in female monkeys with normal cycles. Furthermore, these findings raise the possibility that reinforcing effects of BZ-neuroactive steroid combinations may synergistically enhance the abuse potential of the combinations, making development of low-dose BZ + neuroactive steroid combinations as therapeutic agents potentially untenable, at least in a subset of the patient population.

**Table 4:** Comparison of relative reinforcing potency and effectiveness values between male and female rhesus monkeys trained and maintained on a progressive-ratio schedule of midazolam reinforcement (*N* = 4 per group).

	Triazolam	Pregnanolone
	ED_50_ Mean mg/kg/injection (SEM)	
Male^a^	0.00041 (0.00011)	0.023 (0.009)
Female	0.0018 (0.0007)	0.055 (0.011)
	BP_max_^b^ Mean responses (SEM)	
Male^a^	213 (53)	266 (53)
Female	180 (20)	171 (25)

a Data from male monkeys adapted from Fischer and Rowlett (14).

b BP_max_ is the highest breakpoint obtained, irrespective of dose, with breakpoint determined as last response requirement completed in a progressive-ratio sequence.

The finding that the nature of the interactions between a BZ and a neuroactive steroid may differ between females and males is not surprising, given that sex-specific behavioral and physiological effects of progesterone and neuroactive steroids are well documented (18, 19). In general, women consistently have been documented to be more likely to receive a BZ prescription than men, and to present with substance use disorders involving BZs at higher rates than men [(33); but see (34)], a finding that is largely international/cross-cultural in scope [e.g., (35)]. Preclinical evidence indicates that the effects of BZs can be altered by differences in circulating progesterone levels, e.g., a BZ engendered anxiolytic-like effects during early stages of the estrous cycle, but not the late diestrus phase, in rats (36). These results presumably are tied to differences in endogenous neuroactive steroid levels that are metabolized from progesterone. With respect to the GABA_A_ subtype target site for BZs and neuroactive steroids, little is known about changes in subtype levels and/or constitution across either the menstrual or estrous cycle. However, (37) demonstrated a robust increase in neurons expressing α4, β1, and δ subunits during the late diestrus phase in rat midbrain, whereas levels of these subunits do not fluctuate across time in male rats. Electrophysiological evidence has accrued linking neuroactive steroid action to the extrasynaptically-located δ-subunit containing GABA_A_ receptor (15). Therefore, it is feasible that enhanced action at δ-containing GABA_A_ receptors by exogenous (and/or endogenous) neuroactive steroids may underly enhanced behavioral effects of BZ-neuroactive steroid combinations.

At present, we do not have direct evidence that the supra-additive effects of BZs and pregnanolone in self-administration was linked with progesterone metabolism. Although three of four monkeys exhibited normal cycles based on menstruation, the design of this study, in which drug vs. saline were available across days in a random order, did not allow for accurate tracking of progesterone (and estradiol) levels across the cycle. Specifically, the design of Fischer and Rowlett (14) involved randomized, single-day tests of drugs or drug combinations, counterbalanced in such a way as to reduce the influence of time factors. Nevertheless, systematic evaluation of the effects of neuroactive steroids on BZ self-administration over the course of the menstrual cycle is needed information, as well as conducting studies to more directly assess the role of progesterone (e.g., administration of 5α-reductase inhibitors to block neuroactive steroid formation).

Another goal of the present study was to expand the assessment of combinations of BZs and neuroactive steroids to sedative-motor effects, a prominent adverse effect of GABA_A_ modulators in general. When tested alone, both triazolam and pregnanolone had marked effects on measures of deep sedation and observable ataxia, consistent with other findings using our quantitative observation methods (20, 21). Regarding deep sedation, combination of triazolam and pregnanolone resulted in leftward shifts in the dose-response function for triazolam that tended to not be proportion-dependent. Of the three ratios tested, two resulted in robust supra-additive interactions (1:1, 1:3) with the third trending to supra-additivity. Results with observable ataxia were more complex, with a tendency to decrease these effects rather than enhance them. However, this simply may reflect the enhancements in deep sedation “overriding” ataxic effects.

At present, we do not have sedative-motor data for BZ-neuroactive steroid combinations in male animals. Although not a direct measure of sedative-motor effects, we do have results from studies with male subjects involving operant lever-pressing maintained by food presentation. For example, in male rhesus monkeys, dose-dependent suppression of food-maintained responding by triazolam and pregnanolone demonstrated only additive effects when combined (14). However, in male rats trained under a schedule of food reinforcement, triazolam-pregnanolone combinations resulted in supra-additive interactions, a finding also seen with combinations of other BZs and neuroactive steroids (37). Similarly, food-maintained responding in the context of a triazolam discrimination procedure also showed supra-additive effects with triazolam-pregnanolone combinations in male rats (15), suggesting that at least in rats, the ability to respond/exert motor control may be impaired in an supra-additive fashion in males. However, these similarities with the present study should be interpreted with caution: It is important to note that although changes in schedule-controlled behavior may reflect sedative-motor effects, it is impossible to attribute decreases in operant behavior to any specific behavioral effect, i.e., the decreases could also reflect changes in appetite, motivation, associative processes, and so on.

Across the different behavioral procedures, the combinations of triazolam and pregnanolone were assessed using three different proportions, because changes from additivity often depends on the relative proportion of the drugs in the combinations (31). For both self-administration and deep sedation, there was a similar dependency on proportion: the ratios with the highest level of pregnanolone relative to triazolam (i.e., triazolam:pregnanolone = 1:3 and 1:9 for self-administration and deep sedation, respectively) were less likely to demonstrate supra-additivity. A cautionary note regarding this conclusion is that for self-administration, the 1:3 additive effect reflected results from a single monkey (318-01, see Figure 3), whereas for deep sedation, the average effect of the 1:9 proportion trended to supra-additivity, but did not achieve significance due to variability associated with the pregnanolone effect (see Figure 5). Nevertheless, these results suggest that nature of the interaction of, and perhaps the mechanism(s) underlying, triazolam and pregnanolone combinations may depend on the relative proportion of pregnanolone in the mixture, and further reinforce the utility of testing multiple proportions in drug combination studies.

There are several considerations worth noting for further evaluation of the results and conclusions of this study. Clearly, one monkey believed to be in menopause is an insufficient sample size for making strong conclusions about the role of circulating hormones in the combined effects of BZs and neuroactive steroids. More formal manipulations, such as ovariectomy, may provide a clearer test of this hypothesis. Because menopause is tied to aging, a study in younger monkeys with ovariectomies may help to disentangle whether there are simply age-related differences in responsiveness to BZ-neuroactive steroid combinations. Finally, a study designed to assess changes across the menstrual cycle, concomitant with measuring levels of reproductive hormones and/or endogenous neuroactive steroids, remains a high priority.

In Fischer and Rowlett (14), we raised the possibility that if combining BZs and neuroactive steroids resulted in supra-additive interactions with therapeutic endpoints but infra-additive interactions with endpoints related to adverse effects and/or toxicology, then combining these two drug classes at sub-therapeutic doses may be a viable strategy for developing improved pharmacotherapies (e.g., anxiolytics). The results from the present study present a significant challenge to this idea, due to the finding of pronounced supra-additive effects of the combinations in both self-administration and sedative-motor effects. On the other hand, these data collectively point to a potentially significant sex difference in at least the reinforcing effects of GABA_A_ positive modulators. This is especially noteworthy given the recent approvals of neuroactive steroids ganaxolone and brexanolone in the treatment of epilepsy and postpartum depression, respectively (39). Given the existence of pregnanolone and other neuroactive steroids in the CNS, along with their being metabolic products of reproductive hormones, information about these interactions may lead to a better understanding of sex differences underlying responses to these important psychiatric medications.

## Data availability statement

The raw data supporting the conclusions of this article will be made available by the authors, without undue reservation.

## Ethics statement

The animal study was reviewed and approved by University of Mississippi Medical Center’s Institutional Animal Care and Use Committee.

## Author contributions

JC, DP, DR-B, and JR were responsible for the study concept and design, assisted with data analysis and interpretation of findings. JC and DR-B contributed to data acquisition. JC and DP drafted the manuscript. All authors contributed to the article and approved the submitted version.

## Funding

This work was supported by US National Institute of Health grants DA011792, DA043204, and AA029023.

## Conflict of interest

The authors declare that the research was conducted in the absence of any commercial or financial relationships that could be construed as a potential conflict of interest.

## Publisher’s note

All claims expressed in this article are solely those of the authors and do not necessarily represent those of their affiliated organizations, or those of the publisher, the editors and the reviewers. Any product that may be evaluated in this article, or claim that may be made by its manufacturer, is not guaranteed or endorsed by the publisher.
