# Supplementary material for: Behavioral effects of triazolam and pregnanolone combinations: reinforcing and sedative-motor effects in female rhesus monkeys
Source: Front Psychiatry. 2023 May 12;14:1142531. doi: 10.3389/fpsyt.2023.1142531 (PMC10213563; doi:10.3389/fpsyt.2023.1142531)
Supplement: Supplementary file 1 [file Table_1.DOCX]

Supplementary Material

Behavioral Effects of Triazolam and Pregnanolone Combinations: Reinforcing and Sedative-Motor Effects in Female Rhesus Monkeys

Jemma E. Cook, Donna M. Platt, Daniela Rüedi-Bettschen, James K. Rowlett^*^

***Correspondence:**

James K. Rowlett

[jrowlett@umc.edu](mailto:jrowlett@umc.edu)

**Supplementary Tables (starting on next page)**

| **Table S1.** List of behaviors, abbreviations, and definitions for quantitative behavioral observation. | | |
| --- | --- | --- |
| ***Species-Typical Behaviors*** | **Abbreviation** | **Brief Description** |
| Body Spasm | BSP | An involuntary twitch or shudder of the entire body; also “wet dog” shake |
| Cage Shake | CSH | Any vigorous shaking of the cage that may or may not make noise |
| Drink | DRI | Mouth contact to fluid delivery sippers |
| Fear Grimace | FGR | Grin-like facial expression involving the retraction of the lips exposing clenched teeth; may be accompanied by flattened ears, stiff, huddled body posture, screech/chattering vocalizations |
| Forage | FOR | Sweeping and/or picking through wood chip substrate |
| Lip Droop | LDR | Bottom lip drooping, showing bottom teeth |
| Lip Smack | LIP | Pursing the lips and moving them together to produce a smacking sound, often accompanied by moaning |
| Locomotion | LOC | At least two directed steps in the horizontal and/or vertical plane |
| Nose Rub | NRU | Excessive wiping of nose with hand or arm |
| Observable Ataxia | ATX | Any slip, trip, fall, loss of balance. |
| Passive Visual | VIS | Animal is standing or sitting motionless with eyes open |
| Present | PRE | Posture involving presentation of rump, belly, flank, and/or neck to observer or other monkey |
| Scratch | SCR | Vigorous strokes of the hair with fingers or toenails |
| Self-Groom | GRM | Picking, scraping, spreading or licking of an animal’s own hair |
| Stereotypy | STY | Any repetitive, ritualized pattern of behavior that serves no obvious function |
| Tactile/Oral Exploration | TAC | Any tactile or oral manipulation of the cage or environment |
| Threat/Aggress | THR | Multifaceted display involving one or more of the following: Open mouth stare with teeth partially exposed, eyebrows lifted, ears flattened or flapping, rigid body posture, piloerection, attack (e.g., biting, slapping) of inanimate object or other monkey |
| Tremor/jerk | TRJ | A tremor or jerk of a part of the body (e.g., head, two limbs) |
| Vocalization | VOC | Species-typical sounds emitted by monkey (not differentiated into different types) |
| Vomit/retch | VOM | Expulsion of food or fluid through mouth or nose or making the sound or movement of vomiting |
| Yawn | YWN | To open mouth wide and expose teeth |
| ***Sedation Measures*** |  | |
| Rest/Sleep Posture | RSP | Idiosyncratic posture adopted by monkeys during rest or sleep, easily roused; eyes closed <3 s after stimulus |
| Moderate Sedation | MSE | Atypical loose-limbed posture (e.g., propped on the cage by the body or a limb), eyes closed, delayed response to external stimuli (> 3 sec) |
| Deep Sedation | DSE | Atypical loose-limbed posture, eyes closed, does not respond to external stimuli |

| **Table S2.** *Analysis of all behaviors following multiple doses of triazolam, pregnanolone, and triazolam-pregnanolone combinations.*  *Significant effects were determined as at least one dose significantly different from vehicle (N=4 monkeys, p < .05) and identified with red text.* | | | |
| --- | --- | --- | --- |
| **Drug** | **No Change** | **Increase** | **Decrease** |
| Triazolam | BSP, CSH, DRI, FGR, GRM, LDP, LIP, LOC, MSE, NRU, PRE, RSP, SCR, STY, TAC, THR, TRJ, VIS, VOC, VOM, YWN | ATX, DSE | FOR |
| Pregnanolone | BSP, CSH, DRI, FGR, FOR, GRM, LDP, LIP, LOC, MSE, NRU, PRE, RSP, SCR, STY, TAC, THR, TRJ, VIS VOC, VOM, YWN, | ATX, DSE | -- |
| 1:1 Combination | BSP, CSH, DRI, FGR, FOR, GRM, LDP, LIP, LOC, MSE, NRU, PRE, RSP, SCR, STY, TAC, THR TRJ, VIS VOC, VOM, YWN, | ATX, DSE | -- |
| 1:3 Combination | ATX, BSP, CSH, DRI, FGR, FOR, LDP, LIP, LOC, MSE. NRU, PRE, RSP, SCR, STY, TAC, THR, TRJ, VIS, VOC, VOM, YWN | DSE | SCR, GRM |
| 1:9 Combination | ATX, BSP, CSH, DRI, FGR, FOR, LDP, LIP, MSE, NRU, PRE, RSP, SCR, STY, TAC, THR, TRJ, VIS, VOC, VOM, YWN | DSE | GRM |

| **Table S3.** Actual doses used for the dose combinations in self-administration studies. | | | | |
| --- | --- | --- | --- | --- |
|  |  | **Fixed Dose Ratio (Triazolam:Pregnanolone)** | | |
| **Monkey ID** |  | **1:0.3** | **1:1** | **1:3** |
| 143-03 |  | *Actual Ratio (Triazolam:Pregnanolone)** | | |
|  |  | 1:5 | 1:16 | 1:48 |
|  | Triazolam (mg/kg/injection) | Pregnanolone (mg/kg/injection) | | |
|  | 0.0003 | 0.0015 | 0.0048 | 0.014 |
|  | 0.001 | 0.005 | 0.016 | 0.048 |
|  | 0.003 | 0.015 | 0.048 | 0.14 |
|  | 0.01 | 0.05 | 0.16 |  |
|  | 0.03 | 0.15 |  |  |
|  |  |  |  |  |
| 318-01 |  | *Actual Ratio (Triazolam:Pregnanolone)** | | |
|  |  | 1:11 | 1:33 | 1:100 |
|  | Triazolam (mg/kg/injection) | Pregnanolone (mg/kg/injection) | | |
|  | 0.0003 |  |  | 0.03 |
|  | 0.001 | 0.011 | 0.033 | 0.1 |
|  | 0.003 | 0.033 | 0.099 | 0.3 |
|  | 0.01 | 0.11 | 0.33 | 1 |
|  | 0.03 | 0.33 | 0.99 |  |
|  |  |  |  |  |
| 165-01 |  | *Actual Ratio (Triazolam:Pregnanolone)** | | |
|  |  | 1:12 | 1:36 | 1:109 |
|  | Triazolam (mg/kg/injection) | Pregnanolone (mg/kg/injection) | | |
|  | 0.0003 | 0.0036 |  |  |
|  | 0.001 | 0.012 | 0.036 | 0.109 |
|  | 0.003 | 0.036 | 0.108 | 0.327 |
|  | 0.01 | 0.12 | 0.36 | 1.09 |
|  |  |  |  |  |
| 388-06 |  | *Actual Ratio (Triazolam:Pregnanolone)** | | |
|  |  | 1:16 | 1:49 | 1:146 |
|  | Triazolam (mg/kg/injection) | Pregnanolone (mg/kg/injection) | | |
|  | 0.0003 |  |  | 0.0438 |
|  | 0.001 | 0.016 | 0.049 | 0.146 |
|  | 0.003 | 0.048 | 0.147 | 0.438 |
|  | 0.01 | 0.16 | 0.49 |  |
|  | 0.03 | 0.48 |  |  |
| *Actual Ratio: Proportions used in combinations based on setting the 1:1 ratio equal to the individual ED_50_ values of the drugs alone. | | | | |

| **Table S4.** Actual doses used for the dose combinations in observation studies. | | | |
| --- | --- | --- | --- |
|  | **Fixed Dose Ratio (Triazolam:Pregnanolone)** | | |
|  | **1:1** | **1:3** | **1:9** |
|  | *Actual Ratio (Triazolam:Pregnanolone)** | | |
|  | 1:2 | 1:5 | 1:16 |
| Triazolam (mg/kg/injection) | Pregnanolone (mg/kg/injection) | | |
| 0.01 | 0.02 | 0.05 | 0.16 |
| 0.03 | 0.06 | 0.15 | 0.48 |
| 0.1 | 0.2 | 0.5 | 1.6 |
| 0.3 |  |  | 4.8 |
| *Actual Ratio: Proportions used in combinations based on setting the 1:1 ratio equal to the individual ED_50_ values of the drugs alone. | | | |
